# Genetic risk score for adult body mass index associations with childhood and adolescent weight gain in an African population

**DOI:** 10.1186/s12263-018-0613-7

**Published:** 2018-08-01

**Authors:** Richard J. Munthali, Venesa Sahibdeen, Juliana Kagura, Liesl M. Hendry, Shane A. Norris, Ken K. Ong, Felix R. Day, Zané Lombard

**Affiliations:** 1 Faculty of Science, School of Molecular and Cell Biology, University of the Witwatersrand, Johannesburg, South Africa; 2 Sydney Brenner Institute for Molecular Bioscience (SBIMB), University of the Witwatersrand, The Mount, 9 Jubilee Road, Parktown, Johannesburg, Gauteng 2193 South Africa; 3 MRC/Wits Developmental Pathways for Health Research Unit (DPHRU), University of the Witwatersrand, Johannesburg, South Africa; 4 Faculty of Health Sciences, Division of Human Genetics, School of Pathology, University of the Witwatersrand and National Health Laboratory Service, Johannesburg, South Africa; 5 MRC Epidemiology Unit, Institute of Metabolic Science, University of Cambridge, Cambridge, UK

**Keywords:** Childhood adiposity, Obesity, Genetic risk score, Mediation analysis, Weight gain, Body mass index

## Abstract

**Background:**

Ninety-seven independent single nucleotide polymorphisms (SNPs) are robustly associated with adult body mass index (BMI kg/m^2^) in Caucasian populations. The relevance of such variants in African populations at different stages of the life course (such as childhood) is unclear. We tested whether a genetic risk score composed of the aforementioned SNPs was associated with BMI from infancy to early adulthood. We further tested whether this genetic effect was mediated by conditional weight gain at different growth periods. We used data from the Birth to Twenty Plus Cohort (Bt20+), for 971 urban South African black children from birth to 18 years. DNA was collected at 13 years old and was genotyped using the Metabochip (Illumina) array. The weighted genetic risk score (wGRS) for BMI was constructed based on 71 of the 97 previously reported SNPs.

**Results:**

The cross-sectional association between the wGRS and BMI strengthened with age from 5 to 18 years. The significant associations were observed from 11 to 18 years, and peak effect sizes were observed at 13 and 14 years of age. Results from the linear mixed effects models showed significant interactions between the wGRS and age on longitudinal BMI but no such interactions were observed in sex and the wGRS. A higher wGRS was associated with an increased relative risk of belonging to the early onset obese longitudinal BMI trajectory (relative risk = 1.88; 95%CI 1.28 to 2.76) compared to belonging to a normal longitudinal BMI trajectory. Adolescent conditional relative weight gain had a suggestive mediation effect of 56% on the association between wGRS and obesity risk at 18 years.

**Conclusions:**

The results suggest that genetic susceptibility to higher adult BMI can be tracked from childhood in this African population. This supports the notion that prevention of adult obesity should begin early in life. The genetic risk score combined with other non-genetic risk factors, such as BMI trajectory membership in our case, has the potential to be used to screen for early identification of individuals at increased risk of obesity and other related NCD risk factors in order to reduce the adverse health risk outcomes later.

**Electronic supplementary material:**

The online version of this article (10.1186/s12263-018-0613-7) contains supplementary material, which is available to authorized users.

## Background

Obesity, defined as having a (BMI) ≥ 30 kg/m^2^ in adults, is a growing public health concern globally, including in Africa [1, 2]. Obesity is a complex multifactorial condition with both environmental and genetic factors playing a role. The prevalence of obesity varies widely across Africa ranging from 5.3% in Uganda to 30% in Nigeria and 45.7% in South Africa [2]. Genetic factors contribute about 40–70% of inter-individual variability in BMI [3, 4], in addition to differences in environment and nutritional transitional stages playing a role in variability. Results from the largest and most recent meta-analysis of genome wide association studies (GWAS) identified 97 independent loci that influence adult BMI. The meta-analysis involved approximately 339,224 individuals of predominantly European ancestry from 125 studies [5]. Most of studies of the genetics of BMI focus on effects in adults and the role of genetics at other stages of the life course, especially childhood, are not well studied. However, 15 genetic loci have been reported for BMI in childhood and most of these also influence adult BMI [5–8].

Genetic associations seen in European populations are inconsistently replicated in African and Asian populations [9–12]. Some studies have been conducted in African-American populations, but very few have been performed on the diverse populations within Africa, and even fewer studies using longitudinal data have been conducted [9, 13–15]. Exploring genetic variations in non-European ancestry populations may provide insights not only into establishing the exact risk variants but also into novel risk candidates. Repeat measurements and longitudinal data may enhance our understanding of the timing of such genetic influences, and in particular, life course studies of weight gain and adiposity may identify age-specific determinants [16].

In a recent systematic review of genetic studies for BMI in Africans, Yako and colleagues [12] showed that more than 300 SNPs in 42 genes have been investigated but very few positive findings were replicable. Although 36 of these 300 variants were validated through GWAS in other non-African populations, only one GWAS has been done in continental Africans and a handful of SNPs in or near *FTO* and *MC4R* have been verified to play a role in BMI variability in black South Africans, Ghanaians, and Nigerians through replication studies [12, 17]. A recently published GWAS in West Africans has reported a novel BMI African specific locus, rs80068415, in SEMA4D gene. The lack of replication may be due, in part, to the variation in genetic architecture between different ancestry populations. A recent study in Samoans reported a variant in the *CREBRF* gene (rs373863828) associated with an increased BMI of 1.36 kg/m^2^ per risk allele, which explained 1.93% of the BMI variance in Samoans. Comparatively, the most robustly associated variant in Europeans (rs1558902 in *FTO* gene) has an effect size of 0.39 kg/m^2^ per risk allele and explains only 0.34% of the variance of BMI in Europeans. The *CREBRF* variant is common in Samoans but very rare in other populations, and was shown to be under positive selection in this population––emphasizing the need for studies in diverse populations [18].

Since most reported GWAS loci individually have small effect sizes, many follow-up studies have explored the role of combined risk allele scores [19]. For example, a study on a rural Gambian population reported that a combined allele score of 28 SNPs was positively associated with BMI and adult weight, whereas no association was observed when testing single SNP [10, 20]. Such combined allele scores allude to the possibility of SNP-SNP interaction influencing disease outcome in such complex diseases. Few studies (both in African and non-African cohorts) have described the longitudinal associations between such combined allele scores on changes in BMI across childhood through to 18 years old [10, 21–24]. Furthermore, there is paucity of research to explore whether pathways related to weight gain at specific stages of early life mediate the associations between SNPs and adult BMI. The aims of this study were to determine the combined influence of known genetic risk loci (combined into a weighted genetic risk score (wGRS) on BMI across infancy and late adolescence in a Black South African cohort. We furthermore explored the possible mediating role of childhood and adolescent weight gain on this genetic effect and also examined the association between the wGRS with previously identified childhood BMI trajectories in this cohort [25].

## Methods

### Study sample

The Birth to Twenty Plus Cohort (Bt20+) is a prospective cohort study among black urban South African children born in 1990. The cohort is focused on the health and development of children born in an urban township of Soweto in Johannesburg, South Africa. Since recruitment, the participants have been contacted regularly and longitudinal data and DNA samples were collected at several time points. Details on the cohort have been described elsewhere [26]. Approval for the Bt20+ study was obtained from the Human Research Ethics Committee of the University of the Witwatersrand, Johannesburg (certificate number M0101556). In addition, this committee approved the use of genetic data for this study under certificate number M1411115. Participants or their parents/caregivers where participants were minor gave informed consent at the beginning of each data collection session throughout the study.

### Genotyping and compilation of a genetic risk score

DNA was extracted using the standard salting-out method [27] from blood samples and genotyped using the Metabochip (Illumina, San Diego, California, USA) at the DNA Technologies Core of the University of California Davis (California, USA) [28]. Data were generated on 196,725 SNPs in 1248 participants before quality control (QC) using PLINK version 1.9 [29]. Sixty-eight thousand nine hundred sixty-one SNPs were excluded due to a missing rate > 2%, minor allele frequency < 1%, or departure from Hardy-Weinberg equilibrium (*P* < 1 × 10^−5^). Subsequently, 272 samples were excluded due to genotype missing rate > 3%, extreme heterozygosity between genotypes (± 3 standard deviations (SD) from the mean), discordant sex assignment, identity by descent (IBD) score >  0.1875, duplicate samples, or a population outlier based on principal component analysis (Additional file 1: Figure S1). Five individuals did not have data on childhood BMI trajectory and were also excluded. The final dataset comprised of 127,764 SNPs in 971 participants.

Of the 97 independent adult BMI-associated SNPs reported by Locke et al. [5], 69 were present in our dataset and we identified proxies (*r*^2^ > 0.8) for a further two SNPs, resulting in 71 SNPs for analysis (Additional file 1: Table S1 and Figure S2). Genotypes at these 71 variant positions were combined to construct a weighted BMI genetic risk score (wGRS). Weighting was performed by multiplying the number of BMI-increasing alleles at each locus (0, 1, or 2) in each individual by the corresponding effect size (in kg/m^2^ per allele) as reported by Locke et al. [5]. In the absence of GWAS meta-analysis data in African populations, we used the BMI effect estimates reported in European ancestry populations. The wGRS was rescaled to standard deviation scores (SDS), *Z*-scores (wGRSz), by calculating (individual wGRS value *minus* population mean wGRS)/population standard deviation wGRS. The *Z*-score transformed wGRS were utilized to account for the variation in number of risk alleles constituting the wGRS [22, 30].

### Anthropometric measures and assessment of growth

Trained research assistants measured birth weight, postnatal weight, and height. Weight was measured using a digital scale to the nearest 0.1 kg with participants in light clothes and without shoes. A wall-mounted stadiometer (Holtain, UK) was used to measure standing height to the nearest 0.1 cm. Weight and height values were collected annually from birth up to 23 years and were used to calculate BMI as weight (kg) divided by height (m) squared.

BMI *Z*-scores were computed using the World Health Organization (WHO) growth standards [31]. When dealing with repeated measures, data collected from the same individual over time, the assumption of homogeneity of covariance should be met, that is the correlations between weight measures for each of the pair of ages (time points) should be equal. This is not usually true for weight since weight is also dependent on linear growth and the weight measures at ages close to each other are highly correlated compared to those far apart. Repeated weight measures violate this assumption resulting in overestimation of estimates [32]. To deal with bias from the high correlation of repeated weight measures, conditional relative weight gain independent of linear growth was calculated and used where appropriate. Conditional relative weight gain was calculated for each growth period as standardized residuals (SR) from sex-specific linear regressions of current weight on prior weights, heights, and current height in infancy (0–2 years), early childhood (2–5 years), mid-childhood (5–8 years), and adolescence (8–15 years) [33, 34].

### Childhood adiposity trajectories

We previously identified three and four distinct adiposity trajectories in 877 boys and 947 girls, respectively, in the same group of Bt20+ black South African children, using latent class growth mixture modeling (LCGMM) [25]. These trajectories were characterized from participants with at least two age-point BMI measures between 5 and 18 years of age. On average, seven longitudinal BMI measures were available per participant.

LCGMM assumes that individuals have different growth trajectories and groups individuals with similar growth trajectories together to form a subgroup or a class [35]. Adiposity trajectory membership was assigned depending on the high probability of belonging to a particular trajectory depending on Bayesian posterior probabilities. The adiposity trajectories depicted BMI growth patterns indicative of early onset obesity of overweight, late onset of obesity or overweight, and those with normal weight. Girls and boys in early onset obesity or overweight adiposity trajectory membership had a higher risk of elevated blood pressure at 18 years old [25]. Trajectory membership was graded by increasing risk of childhood obesity and used as an outcome variable in multinomial logistic regression models with wGRSz as an independent variable. Upon merging data for girls and boys, trajectories with similar patterns were combined, with one trajectory (early onset obese to overweight) comprising of girls only.

### Statistical analyses

Linear, mixed, and multinomial logistic regression models were used to examine the cross-sectional and longitudinal associations between wGRSz and BMI from infancy to early adulthood, adjusting for sex. It was previously reported that the patterns of missing data on weight and height are likely to be missing at random [25]; we still reduced the potential bias by not excluding individuals who did not have data at every time point. The linear mixed-effects models, wGRSz on longitudinal BMI with random intercepts by the participants’ id, were used to find associations between wGRSz and longitudinal BMI. The effects of age and sex interactions with wGRSz on longitudinal BMI were also tested. The interaction between genetic risk score and linear age, (wGRSz*age), quadratic age (wGRSz*age^2^), and the cube of age (wGRSz*age^3^) were calculated to capture both linear and nonlinear growth interactions with age; wGRSz*age interactions can be interpreted as effects of linear change in BMI *Z*-score with age. Mediation analyses decomposed GRS-BMI associations into two parts, direct (unmediated) and indirect (mediated through weight gain at different developmental stages) components. A Sobel test [36] was used to estimate the indirect effect of infancy, childhood, and adolescent growth (conditional relative weight gain) on the association between wGRSz and BMI *Z*-scores in early adulthood (Additional file 1: Figure S3). When doing mediation analysis, both the normal Sobel, with assumption of normal distribution, and Sobel test with bootstrapping were used [37, 38]. The bootstrap option computes the bias-corrected confidence intervals with no distribution assumptions. To correct the problem of multiple testing when comparing the indirect effects, we employed the Holm’s sequential correction, which is less stringent but more powerful than the Bonferroni correction [39].

Since BMI trajectories represent an individual’s life course growth, we were also interested to find out if the genetic risk score could predispose/influence an individual to belong in a particular BMI trajectory group. We estimated the association between wGRSz and BMI trajectory membership using multinomial logistic regressions, and the normal weight adiposity trajectory group was used as a reference for the analysis. All analyses were performed using STATA version 13.0 (STATA Corp, TEXAS). A *P* value < 0.05 was considered statistically significant unless otherwise stated.

## Results

Results from the study characteristics at 18 years showed that average height and BMI were statistically different between boys and girls while weight (59.4 kg in boys and 59.5 kg in girls) was not. Boys were taller (170.8 cm) than girls (159.7 cm) while girls had a higher mean BMI than boys (23.3 vs 20.4 kg/m^2^), see Table 1. The mean BMI growth curves from 5 to 18 years are presented in Additional file 1: Figure S5. Despite having similar mean BMI at 5 years, girls became heavier than boys over time. The number of participants with an increasing number of risk alleles (in the form of wGRSz) followed an approximately normal distribution as expected (Additional file 1**:** Figure S4). Only five of the 71 individual SNPs were associated with BMI at 18 years of age (Additional file 1: Table S3).

**Table 1:** Comparing study characteristics between boys and girls study participants

Variable	Age (years)											
	0		2		5		8		15		18	
	M	F	M	F	M	F	M	F	M	F	M	F
Height (cm)	67.7 (7.3)	66.5 (6.8)	83.7 (3.1)*	82.9 (3.2)	107.6 (4.4)	107.2(4.6)	125.1 (5.7)	124.3 (5.5)	165.5 (7.7)*	158.7 (6.2)	170.8 (6.5)*	159.7 (6.0)
Weight (kg)	3.1 (0.5)*	3.0 (0.5)	11.4 (1.4)*	11.2 (1.3)	18.2 (2.2)	18.0 (2.7)	25.1 (3.7)	25.1 (5.0)	53.7 (10.1)*	56.3 (12.6)	59.4 (9.7)	59.5 (12.9)
BMI (kg/m^2^)	17.7 (2.3)	17.4 (2.2)	16.4 (2.1)	16.3 (1.8)	15.8 (1.3)	15.6	16.0 (1.5)	16.1 (2.4)	19.5 (3.1)*	22.33 (4.8)	20.3 (3.1)*	23.3 (4.8)
BMI *Z*-score	0.4 (1.6)*	0.5 (1.3)	0.2 (1.6)	0.4 (1.2)	0.3 (1.0)*	0.1 (0.9)	− 0.002 (0.9)	− 0.003 (1.0)	− 0.5 (1.1)*	0.3 (1.2)	− 0.7 (1.0)*	0.4 (1.2)
Sample size (*n*)	516	453	346	305	413	356	304	257	476	436	493	429

*M* male, *F* female

*P < 0.05

**Fig. 1:**
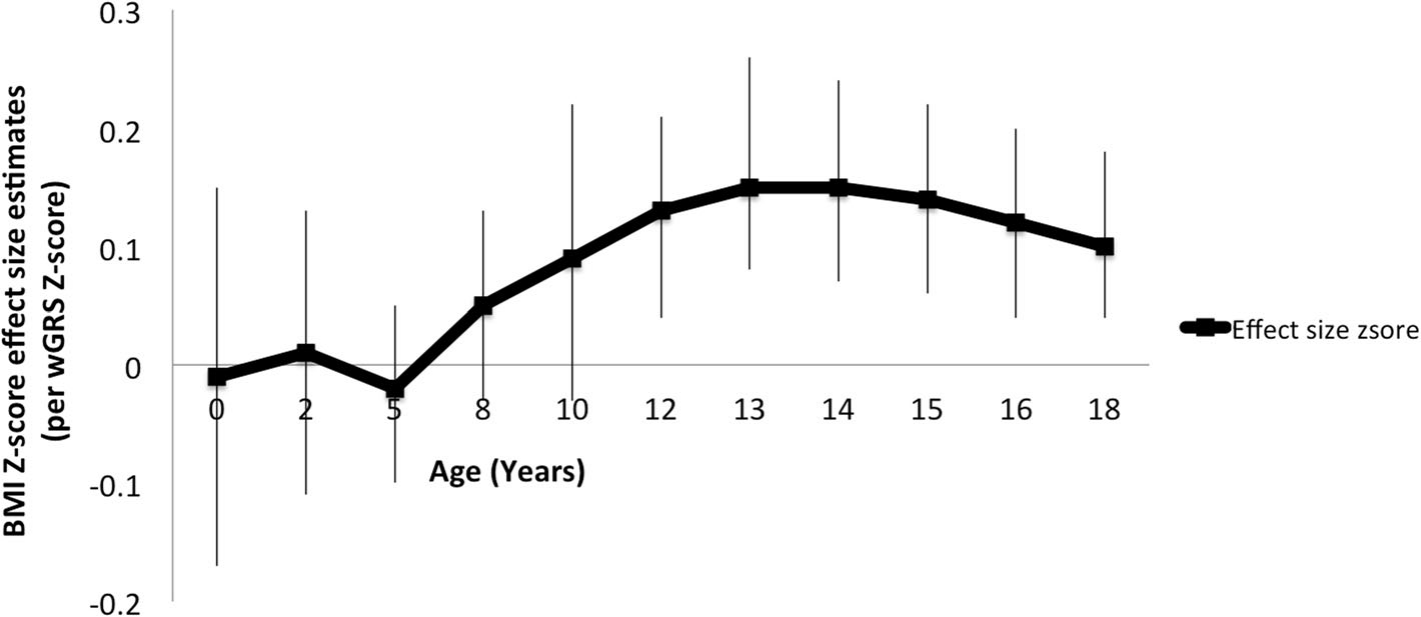
Effect size estimates for BMI *Z*-score per *Z*-score increase in wGRS at each age adjusted for sex (error bars indicate 95% confidence intervals)

Evaluation of the cross-sectional association between wGRSz and BMI *Z*-score showed that the effect size estimates increased in magnitude with age until the age of 14 years. Significant associations were observed from ages 11 through to 18 years (Fig. 1). For each additional standard deviation increase, wGRSz was associated with a 0.15 SD increase in BMI *Z*-score. This was observed at years 13 and 14. The wGRSz was not associated with birth weight.

Results from the linear mixed effects models; significant interactions between age and wGRSz on BMI SDS were observed for linear age “age” *(β* = 0.01 SDS per SD per year; 95% CI 0.006 to 0.012), quadratic age “age^2^” (*P* < 0.001), and age cubic “age^3^” (*P* < 0.001) terms, but there was no such interaction with “sex.” The multinomial logistic regression results showed that wGRSz positively predicted membership of the previously reported “early onset obese BMI trajectory” compared to those in a “normal BMI trajectory” [25] (relative risk ratio = 1.88; 95% CI 1.28 to 2.76).

### Mediation by childhood and adolescent weight gain

**Fig. 2:**
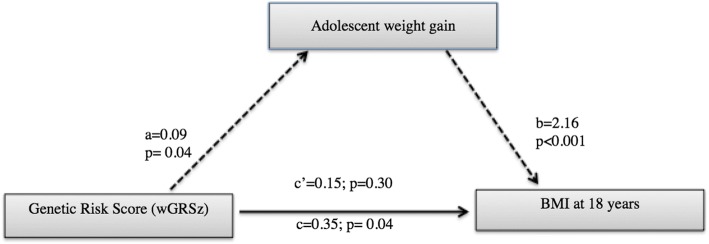
Impact of weighted genetic risk score (wGRSz) on BMI at 18 years via adolescent conditional relative weight gain. Notes: Path *a* is the direct effect of the wGRSz on mediator (adolescent weight gain). Path *b* is the mediator’s effect on the outcome (BMI at 18 years) eliminating the effect of wGRSz. The total effect of wGRSz on BMI at 18 years is *c* = *c*’ + *a* × *b*, where *a* × *b* is the indirect effect of wGRSz on BMI at 18 years via adolescent weight gain

The Sobel test showed a suggestive mediating effect of conditional relative weight gain during adolescence (56% of total effect, uncorrected *P* = 0.037 < 0.05) on the association between the wGRSz and BMI at 18 years, the pathway diagram is shown in Fig. 2. This association disappears after adjusting for the Holm’s sequential Bonferroni multiple testing correction [39], *P* = 0.037 > 0.0125. Although not significant, it is interesting to note that mid-childhood conditional relative weight gain mediated a further 28% of this association (uncorrected *P* = 0.15). Conditional relative weight gain in infancy (0.02% of total effect mediated) and early childhood (1.7% of total effect mediated) did not appear to mediate the wGRSz association with adult BMI as evidenced in the significant corrected *P* values of the direct effect of wGRSz on BMI at 18 years old (Additional file 1: Table S2). The confidence intervals from the Sobel with sample and residual resampling through bootstrapping method confirmed these results.

## Discussion

In an African population, followed longitudinally from birth to 18 years, we explored the cumulative effect of adult BMI-associated risk variants identified in Caucasian samples on the trajectory of childhood BMI. Only five single SNPs of the 71 previously reported to be associated with adult BMI were replicated in the current study, this is of no surprise, because our sample size (*n* = 971) was significantly smaller than that used in the GWAS meta-analysis for the discovery of these SNPs (*n* = 339, 224) [5]. The small sample size may have also influenced the suggestive significant and non-significant indirect effects of the conditional relative weight gain at different growth periods on the wGRS-BMI at 18 years association observed in the current study. Our results also show that the genetic variants that influence adult BMI become increasingly important during the adolescent years (11 to 18 years, peaking between 13 and 14 years). This suggests that the genetic influence on the etiology of obesity follows an increasingly adult pattern starting at age 11 (and possibly earlier in other cohorts). The wGRSz was not associated with BMI increase in infancy and early childhood, indicating that the current cumulative risk score is perhaps not relevant during this period of development. It might also reflect that rapid weight gain in infancy and early childhood are unlikely pathways through which genetic risk for obesity manifests, but other pathways during these growth periods cannot be ruled out [40]. The observation that there was no significant interaction between the genetic score and sex suggests that the genetic etiology of obesity is not substantially different between the sexes, despite the observed differences in BMI growth trajectories [25]. In South Africa, obesity prevalence is much higher in women (70%) than men (40%) across the life course, and further investigation in this sex-specific observation is needed [2, 41].

Only one previous study utilized wGRS on longitudinal BMI in childhood in Africans, which reported that a genetic risk score was associated with adult weight but not with birth weight, similar to what was observed in this current study [10]. Similarly, a non-significant association between GRS and birth weight has also been previously reported in a non-African population [42]. The effect of a wGRS on BMI increased linearly with age in the Gambian population, which is also in line with our current findings [10]. Similar study designs have been reported in European populations. Among 3975 Dutch children with a median age of 6 years, the peak effect of the genetic score on BMI was observed at age 6, [22] which contrasts with the non-significant inverse trend with BMI at age 5 in our current study. In 9328 British and Australian children, a GRS of 32 adult BMI-associated SNPs was positively associated with BMI changes from after birth to 16 years [42]. Another study in 652 Norwegian children, followed from age 4 years through age 8 years, reported that a similar 32-SNP GRS was associated with accelerated childhood weight gain during this period, although appetite traits did not appear to mediate these associations [43]. In 5906 Native Americans of predominantly Pima Indian heritage aged between 5 and 45 years, 36 SNPs were associated with childhood BMI and allelic risk score, and a subset from these 36 SNPs was associated with increased birth weight and greater rates of BMI change in childhood [44]. These results might reflect differences in the obesogenic environments between children in different parts of the globe, especially as it is thought that large energy surpluses in early life could result in earlier puberty and adult development [45]. We cannot rule out the possibility that the GRS may mediate earlier effects on weight gain and BMI changes in more obesogenic settings. There is consistent evidence that genetic susceptibility to higher adult BMI promotes faster weight gain in childhood in different populations, but specific influences (perhaps related to local environments) may determine the onset and peak timing of these effects [42].

Other factors might contribute to the apparent differences in the timing of effects of GRS on weight gain between Caucasian and African population, in particular the marked differences in genetic architecture among these populations [46]. It has also been suggested that such differences could contribute to the inconsistent replication of BMI-associated loci in black African populations [9, 10, 12, 20]. Furthermore, the role of epigenetics during this critical growth period cannot be ruled out [40, 47, 48]. The possible mechanisms in which DNA methylation, histone and chromatin modification, and post-translational regulation might influence obesity in childhood and adolescence have been explored in several studies. It has also been suggested that exercise and diet during this period could help to counter some of the epigenetics effects [49–51].

There has been an increasing interest in understanding factors influencing the variation in BMI growth patterns from childhood to adulthood. Our current findings have shown that a higher wGRSz puts an individual at increased risk of belonging to a growth trajectory characterized by high BMI values across childhood and adolescence. This is consistent with results from a recent study which reported that individuals in a heavy weight BMI trajectory were characterized having a higher GRS, constructed from 97 SNPs based on the same GWAS study as the 71 SNPs utilized in our current study, compared to those belonging lean and medium BMI trajectories from 5 to 65 years in 7277 and 4645 American women and men, respectively [52]. We also observed significant associations from the cross-sectional association between wGRSz and BMI from late childhood to adolescent period, but there is clearly a role for other factors––early life growth, socio-demographic, socioeconomic, ethnicity, and gender [53–56]. There may also be genetic factors that are specific to this age range: a recent study in 1229 Mexican American adolescents reported that genetic polymorphisms in genes related to risk-taking behaviors such as those in *COMT*, *HTR2A*, and *SLC6A3A* were associated with BMI trajectory membership [57]. As our study considered variants previously associated with adult BMI, discovery of these types of effects was beyond the scope of the current study, but genome-wide studies of adiposity at these ages may reveal different SNPs that are associated with adiposity biology. In particular, using defined growth trajectories as the outcome of interest has the specific benefit of reducing modeling complexity in longitudinal genetic data analysis. This method has a potential clinical significance, as it would be able to pinpoint the most at risk subgroups (based on their GRS), and could be utilized to predict early disease onset and progression based on the trajectory membership [58, 59].

The main strength of our study is the use of longitudinal data and different longitudinal methods to understand the influence of the genetic risk score on BMI. Our study demonstrates the likely utility of modeling BMI trajectories in future genetic studies in large sample sizes. This approach would also allow harmonization between studies that collected data at different time points.

The study has a few weaknesses. Firstly, only a single African population was studied here meaning that the generalizability of our findings to other African populations will need to be determined.

Secondly, the BMI genetic risk score was generated from predominantly Caucasian studies; the wGRSz constructed may not be a true reflection of BMI genetic risk in African populations. There is a need for large scale GWAS data in African populations as well as meta-analyses of GWAS of obesity in Africa. This would help to establish variants from an African perspective for constructing a GRS. Recently, deliberate efforts have been made to help understand genetic variation within African populations and across other ancestries and also to understand the genetic and environmental factors to complex disease susceptibility through initiatives such as the Southern African Human Genome Project [60], the African Genome Variation Project [61], and the National Institutes of Health (NIH) and Wellcome Trust funded Human Heredity and Health in Africa (H3Africa) Consortium [62]. Data from these projects and others will be vital in genetic studies in continental African populations. Since BMI is influenced by both genetic and environmental factors, our predictions might have been overestimated or underestimated since some potential confounders such as maternal pre-pregnancy BMI and other maternal factors were unavailable and were not considered in the current study. Finally, the genetic risk score approach gives us an insight into the overall genetic susceptibility but much larger studies will be required to examine effects of specific individual genes.

## Conclusions

In conclusion, we observed a significant combined effect of adult BMI-associated genetic variants on childhood and adolescent BMI and childhood adiposity trajectories in a black South African population. Therefore, the adult BMI-associated SNPs in Caucasian population collectively have relevance effect on BMI or obesity throughout the life course in an African population. There seems to be some transferability of these “Caucasian” adult obesity risk SNPs to an African cohort, even in lieu of direct association testing. These findings also suggest that a genetic risk score combined with BMI trajectory membership has the potential to help improve the screening process of individuals to be targeted in coming up with targeted educational and behavior intervention programs for obesity. These programs should target individuals at risk at early stage in order to reduce the adverse health risk outcomes later.

## Additional file

Additional file 1: **Table S1.** Details of the 71 SNPs used to construct the weighted BMI genetic risk score. **Table S2.** Path coefficients, direct and total effect estimates: The role of conditional relative weight gain as a mediator of the association between the weighted genetic risk score (wGRS) and BMI at 18 years of age. **Table S3.** Association between the 71 SNPs and BMI at 18 years. **Figure S1.** Principal component analysis plot comparing the Birth to Twenty Plus participants’ genetic variation to various African populations following quality control. A total of 83% of genetic variation is captured by PC 1 (60.5%) and PC 2 (22.5%). **Figure S2.** Flowchart displaying the selection of BMI related SNPs for inclusion in the weighted BMI genetic risk score. **Figure S3.** Schematic diagram for the mediation analysis to assess whether the association between wGRS and obesity risk at 18 years was mediated by growth. **Figure S4.** Distribution of the weighted genetic risk score for adult BMI in the Birth to Twenty Plus cohort. **Figure S5.** The average BMI (standard deviation bars) values from 5 to 18 years in the Birth to Twenty Plus cohort. (DOCX 288 kb)

## Abbreviations

BMI: Body mass index
COMT: Catechol-O-methyltransferase
CREBRF: CAMP responsive element binding protein 3 regulatory factor
FTO: Fat mass and obesity-associated protein
HTR2A: 5-hydroxytryptamine receptor 2A
LCGMM: Latent class growth mixture modeling
MC4R: Melanocortin 4 receptor
SLC6A3A: Solute carrier family 6 (neurotransmitter transporter, dopamine), member 3
SNP: Single nucleotide polymorphism
wGRS: Weighted genetic risk score
