# Supplementary material for: Genetic risk score for adult body mass index associations with childhood and adolescent weight gain in an African population
Source: Genes Nutr. 2018 Aug 1;13:24. doi: 10.1186/s12263-018-0613-7 (PMC6090951; doi:10.1186/s12263-018-0613-7)
Supplement: Supplementary file 1 — Table S1. Details of the 71 SNPs used to construct the weighted BMI genetic risk score. Table S2. Path coefficients, direct and total effect estimates: The role of conditional relative weight gain as a mediator of the association between the weighted genetic risk score (wGRS) and BMI at 18 years of age. Table S3. Association between the 71 SNPs and BMI at 18 years. Figure S1. Principal component analysis plot comparing the Birth to Twenty Plus participants’ genetic variation to various African populations following quality control. A total of 83% of genetic variation is captured by PC 1 (60.5%) and PC 2 (22.5%). Figure S2. Flowchart displaying the selection of BMI related SNPs for inclusion in the weighted BMI genetic risk score. Figure S3. Schematic diagram for the mediation analysis to assess whether the association between wGRS and obesity risk at 18 years was mediated by growth. Figure S4. Distribution of the weighted genetic risk score for adult BMI in the Birth to Twenty Plus cohort. Figure S5. The average BMI (standard deviation bars) values from 5 to 18 years in the Birth to Twenty Plus cohort. (DOCX 288 kb) [file 12263_2018_613_MOESM1_ESM.docx]

| **Table S1: Details of the 71 SNPs used to construct the weighted BMI genetic risk score** | | | | | | | | | | | | | | | | | | | | | |  |  |  |
| --- | --- | --- | --- | --- | --- | --- | --- | --- | --- | --- | --- | --- | --- | --- | --- | --- | --- | --- | --- | --- | --- | --- | --- | --- |
| **SNP** | **Chr.** | **Position (bp/hg19)** | | **Nearest gene** | | **Effect Allele** | **Other**  **Allele** | | **A1**  **(minor_btt20+)** | **Minor (all_1000g)** | | | **CEU allele freq** | | | **YRI allele freq** | | | **Locke effect**  **allele freq** | | |  |  |  |
| rs1000940 | 17 | 5,223,976 | | *RABEP1* | | G | A | | G | | | G | | 0.35 | | | 0.23 | | | 0.32 | | |  |  |
| rs10408163(1) | 19 | 52,260,843 | | *ZC3H4* | | A | G | | G | | | G | | 0.69 | | | 0.10 | | | 0.65 | | |  |  |
| rs10733682 | 9 | 128,500,735 | | *LMX1B* | | A | G | | A | | | G | | 0.51 | | | 0.74 | | | 0.48 | | |  |  |
| rs10968576 | 9 | 28,404,339 | | *LINGO2* | | G | A | | G | | | G | | 0.31 | | | 0.15 | | | 0.32 | | |  |  |
| rs11126666 | 2 | 26,782,315 | | *KCNK3* | | A | G | | A | | | A | | 0.28 | | | 0.17 | | | 0.28 | | |  |  |
| rs11583200 | 1 | 50,332,407 | | *ELAVL4* | | C | T | | T | | | T | | 0.63 | | | 0.28 | | | 0.41 | | |  |  |
| rs1167827 | 7 | 75,001,105 | | *HIP1* | | G | A | | A | | | G | | 0.54 | | | 0.95 | | | 0.56 | | |  |  |
| rs11688816 | 2 | 62,906,552 | | *EHBP1* | | G | A | | A | | | A | | 0.53 | | | 0.35 | | | 0.53 | | |  |  |
| rs11847697 | 14 | 29,584,863 | | *PRKD1* | | T | C | | T | | | T | | 0.05 | | | 0.38 | | | 0.10 | | |  |  |
| rs12286929 | 11 | 114,527,614 | | *CADM1* | | G | A | | A | | | G | | 0.49 | | | 0.59 | | | 0.52 | | |  |  |
| rs12401738 | 1 | 78,219,349 | | *FUBP1* | | A | G | | A | | | A | | 0.32 | | | 0.09 | | | 0.35 | | |  |  |
| rs12446632 | 16 | 19,842,890 | | *GPRC5B* | | G | A | | A | | | A | | 0.12 | | | 0.09 | | | 0.87 | | |  |  |
| rs12566985 | 1 | 74,774,781 | | *FPGT-TNNI3K* | | G | A | | A | | | A | | 0.53 | | | 0.17 | | | 0.46 | | |  |  |
| rs12940622 | 17 | 76,230,166 | | *RPTOR* | | G | A | | G | | | A | | 0.43 | | | 0.60 | | | 0.57 | | |  |  |
| rs13021737 | 2 | 622,348 | | *TMEM18* | | G | A | | A | | | G | | 0.82 | | | 0.93 | | | 0.83 | | |  |  |
| rs13191362 | 6 | 162,953,340 | | *PARK2* | | A | G | | G | | | G | | 0.12 | | | 0.03 | | | 0.88 | | |  |  |
| rs1441264 | 13 | 78,478,920 | | *MIR548A2* | | A | G | | G | | | A | | 0.65 | | | 0.70 | | | 0.61 | | |  |  |
| rs1460676 | 2 | 164,275,935 | | *FIGN* | | C | T | | C | | | C | | 0.19 | | | 0.25 | | | 0.18 | | |  |  |
| rs1516725 | 3 | 187,306,698 | | *ETV5* | | C | T | | T | | | C | | 0.86 | | | 0.82 | | | 0.87 | | |  |  |
| rs1528435 | 2 | 181,259,207 | | *UBE2E3* | | T | C | | C | | | T | | 0.6 | | | 0.63 | | | 0.63 | | |  |  |
| rs1558902 | 16 | 52,361,075 | | *FTO* | | A | T | | A | | | A | | 0.44 | | | 0.05 | | | 0.41 | | |  |  |
| rs16907751 | 8 | 81,538,012 | | *ZBTB10* | | C | T | | T | | | T | | 0.13 | | | 0.06 | | | 0.91 | | |  |  |
| rs16951275 | 15 | 65,864,222 | | *MAP2K5* | | T | C | | C | | | C | | 0.23 | | | 0.42 | | | 0.77 | | |  |  |
| *rs17001561(2)* | 4 | 77,348,592 | | *SCARB2* | | G | A | | A | | | A | | 0.16 | | | 0.12 | | | 0.15 | | |  |  |
| rs17024393 | 1 | 109,956,211 | | *GNAT2* | | C | T | | C | | | C | | 0.04 | | | 0.08 | | | 0.04 | | |  |  |
| rs17405819 | 8 | 76,969,139 | | *HNF4G* | | T | C | | C | | | C | | 0.29 | | | 0.03 | | | 0.70 | | |  |  |
| rs17724992 | 19 | 18,315,825 | | *PGPEP1* | | A | G | | G | | | G | | 0.27 | | | 0.08 | | | 0.74 | | |  |  |
| rs1808579 | 18 | 19,358,886 | | *C18orf8* | | C | T | | T | | | T | | 0.46 | | | 0.45 | | | 0.53 | | |  |  |
| rs1928295 | 9 | 119,418,304 | | *TLR4* | | T | C | | C | | | C | | 0.43 | | | 0.44 | | | 0.55 | | |  |  |
| rs2033732 | | 8 | | 85,242,264 | | *RALYL* | C | | T | T | | C | | | | 0.76 | | | 0.9 | | | 0.75 | | |
| rs205262 | | 6 | | 34,671,142 | | *C6orf106* | G | | A | A | | G | | | | 0.27 | | | 0.72 | | | 0.29 | | |
| rs2075650 | | 19 | | 50,087,459 | | *TOMM40* | A | | G | G | | G | | | | 0.13 | | | 0.17 | | | 0.85 | | |
| rs2080454 | | 16 | | 47,620,091 | | *CBLN1* | C | | A | A | | A | | | | 0.61 | | | 0.32 | | | 0.41 | | |
| rs2112347 | | 5 | | 75,050,998 | | *POC5* | T | | G | G | | G | | | | 0.38 | | | 0.51 | | | 0.62 | | |
| rs2121279 | | 2 | | 142,759,755 | | *LRP1B* | T | | C | T | | T | | | | 0.13 | | | 0.02 | | | 0.15 | | |
| rs2176040 | | 2 | | 226,801,046 | | *LOC646736* | A | | G | A | | G | | | | 0.63 | | | 0.67 | | | 0.36 | | |
| rs2176598 | | 11 | | 43,820,854 | | *HSD17B12* | T | | C | T | | C | | | | 0.75 | | | 0.62 | | | 0.26 | | |
| rs2207139 | | 6 | | 50,953,449 | | *TFAP2B* | G | | A | G | | G | | | | 0.18 | | | 0.07 | | | 0.18 | | |
| rs2245368 | | 7 | | 76,446,079 | | *PMS2L11* | C | | T | C | | T | | | | 0.79 | | | 0.82 | | | 0.19 | | |
| rs2287019 | | 19 | | 50,894,012 | | *QPCTL* | C | | T | T | | T | | | | 0.19 | | | 0.12 | | | 0.81 | | |
| rs2365389 | | 3 | | 61,211,502 | | *FHIT* | C | | T | C | | T | | | | 0.40 | | | 0.85 | | | 0.57 | | |
| rs2820292 | | 1 | | 200,050,910 | | *NAV1* | C | | A | C | | C | | | | 0.55 | | | 0.38 | | | 0.55 | | |
| rs2836754 | | 21 | | 39,213,610 | | *ETS2* | C | | T | C | | C | | | | 0.60 | | | 0.29 | | | 0.60 | | |
| rs29941 | | 19 | | 39,001,372 | | *KCTD15* | G | | A | A | | G | | | | 0.68 | | | 0.86 | | | 0.67 | | |
| rs3101336 | | 1 | | 72,523,773 | | *NEGR1* | C | | T | C | | C | | | | 0.63 | | | 0.53 | | | 0.61 | | |
| rs3736485 | | 15 | | 49,535,902 | | *DMXL2* | A | | G | G | | G | | | | 0.54 | | | 0.39 | | | 0.46 | | |
| rs3817334 | | 11 | | 47,607,569 | | *MTCH2* | T | | C | T | | T | | | | 0.42 | | | 0.23 | | | 0.40 | | |
| rs3888190 | | 16 | | 28,796,987 | | *ATP2A1* | A | | C | A | | A | | | | 0.34 | | | 0.20 | | | 0.40 | | |
| rs4256980 | | 11 | | 8,630,515 | | *TRIM66* | G | | C | G | | G | | | | 0.63 | | | 0.51 | | | 0.64 | | |
| rs4740619 | | 9 | | 15,624,326 | | *C9orf93* | T | | C | C | | C | | | | 0.48 | | | 0.50 | | | 0.54 | | |
| rs4787491 | | 16 | | 29,922,838 | | *INO80E* | G | | A | G | | G | | | | 0.53 | | | 0.53 | | | 0.51 | | |
| rs543874 | | 1 | | 176,156,103 | | *SEC16B* | G | | A | G | | G | | | | 0.20 | | | 0.28 | | | 0.20 | | |
| rs6465468 | | 7 | | 95,007,450 | | *ASB4* | T | | G | T | | T | | | | 0.30 | | | 0.11 | | | 0.30 | | |
| rs6477694 | | 9 | | 110,972,163 | | *EPB41L4B* | C | | T | T | | T | | | | 0.65 | | | 0.59 | | | 0.37 | | |
| rs6567160 | | 18 | | 55,980,115 | | *MC4R* | C | | T | C | | C | | | | 0.23 | | | 0.24 | | | 0.24 | | |
| rs657452 | | 1 | | 49,362,434 | | *AGBL4* | A | | G | G | | G | | | | 0.63 | | | 0.44 | | | 0.40 | | |
| rs6804842 | | 3 | | 25,081,441 | | *RARB* | G | | A | G | | G | | | | 0.57 | | | 0.37 | | | 0.57 | | |
| rs7138803 | | 12 | | 48,533,735 | | *BCDIN3D* | A | | G | A | | A | | | | 0.34 | | | 0.18 | | | 0.38 | | |
| rs7141420 | | 14 | | 78,969,207 | | *NRXN3* | T | | C | C | | T | | | | 0.52 | | | 0.58 | | | 0.53 | | |
| rs7164727 | | 15 | | 70,881,044 | | *LOC100287559* | T | | C | T | | T | | | | 0.68 | | | 0.30 | | | 0.67 | | |
| rs7239883 | | 18 | | 38,401,669 | | *LOC284260* | G | | A | A | | A | | | | 0.58 | | | 0.53 | | | 0.39 | | |
| rs7243357 | | 18 | | 55,034,299 | | *GRP* | T | | G | G | | G | | | | 0.19 | | | 0.15 | | | 0.81 | | |
| rs7599312 | | 2 | | 213,121,476 | | *ERBB4* | G | | A | A | | A | | | | 0.26 | | | 0.38 | | | 0.72 | | |
| rs7715256 | | 5 | | 153,518,086 | | *GALNT10* | G | | T | G | | T | | | | 0.57 | | | 0.66 | | | 0.42 | | |
| rs7899106 | | 10 | | 87,400,884 | | *GRID1* | G | | A | G | | G | | | | 0.04 | | | 0.16 | | | 0.06 | | |
| rs7903146 | | 10 | | 114,748,339 | | *TCF7L2* | C | | T | T | | T | | | | 0.31 | | | 0.28 | | | 0.71 | | |
| rs9374842 | | 6 | | 120,227,364 | | *LOC285762* | T | | C | C | | T | | | | 0.73 | | | 0.79 | | | 0.75 | | |
| rs9400239 | | 6 | | 109,084,356 | | *FOXO3* | C | | T | C | | C | | | | 0.66 | | | 0.21 | | | 0.67 | | |
| rs977747 | | 1 | | 47,457,264 | | *TAL1* | T | | G | G | | G | | | | 0.61 | | | 0.29 | | | 0.40 | | |
| rs9914578 | | 17 | | 1,951,886 | | *SMG6* | G | | C | C | | G | | | | 0.21 | | | 0.60 | | | 0.23 | | |
| rs9925964 | | 16 | | 31,037,396 | | *KAT8* | A | | G | G | | G | | | | 0.40 | | | 0.09 | | | 0.62 | | |

**Notes**

**SNP:** Single nucleotide polymorphism

**Chr:** Chromosome

**Effect Allele:** The effect allele as reported by Locke et al. (2015)

**Other Allele:** Other allele as reported by Locke et al. (2015)

**A1 (minor_btt20+):** Minor allele in the current study, from the Bt20+ dataset

**Minor (all_1000g):** Minor allele as reported in the 1000 Genomes dataset

**CEU allele freq:** Reference European allele frequency from 1000 Genomes (obtained from Locke et al. 2015)

**YRI allele freq:** African allele (Yoruba) frequency of the effect allele (as per Locke et al. 2015) from 1000 Genomes dataset

**Locke effect allele freq:** Allele frequency of the effect allele in Locke et al. (2015) dataset

**rs10408163 (1)** proxy SNP for rs3810291 from Locke et al. (2015) dataset

**rs17001561 (2)** proxy SNP for rs17001654 from Locke et al. (2015) dataset

**Population Structure and Principal Component Analysis (PCA)**

PCA was used to identify population structure and outliers as part of the QC. Supplemental Figure 4 show PC plot following the removal of outliers in the previous steps as explained in the main manusript (page). The Birth to Twenty Plus Cohort (Bt20+) samples clusters together. PC plot from the first two components were drawn using 12100 SNPs. The Bt20 (red triangles) strongly overlaps with previously studied Bt20 participants (black Sowetans-BSO) (green squares) and southeastern Bantu-speakers (blue circles). The samples cluster distinctly from some African 1000G samples but seem to share closer ancestry with the Luhya (LWK, blue triangles) and Yoruba (YRI, purple triangles). The samples also share some ancestry with other Southern Africa populations, the Herero (SWB) from Botswana and Namibia (yellow squares) as illustrated by the close clustering in Supplimental Figure 3.

***
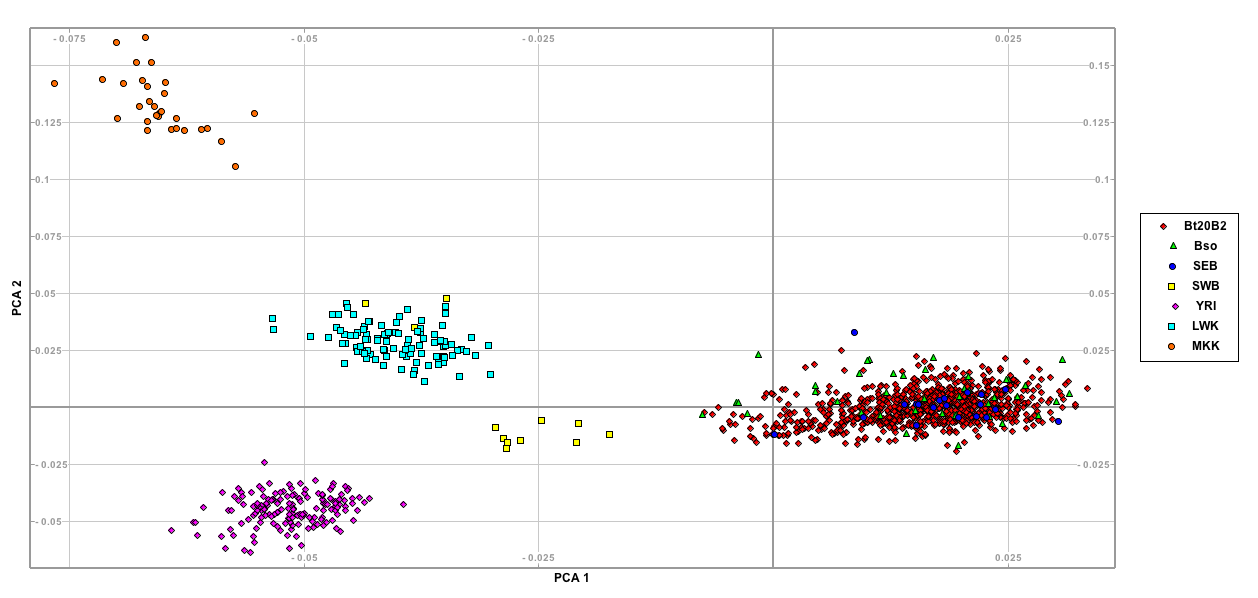
***

**Figure S1:** Principal component analysis plot comparing the Birth to Twenty Plus participants’ genetic variation to various African populations following quality control. A total of 83% of genetic variation is captured by PC 1 (60.5%) and PC 2 (22.5%).

Southeastern Bantu languages belong to the Niger-Congo (Niger-Kordofanian) ethno-linguistic group, being one of the four major language groups spoken in Africa (1), along with Afro-asiatic, Nilo-Saharan and Khoe (2). The Yoruban (YRI, west African) and Luhya (LWK, east African) individuals from the 1000 Genomes Project (3) all speak Niger-Congo languages, which contribute to their relatively close clustering with the Bt20+ samples. The Masaai (MKK) from Kenya (east African), who speak a Nilo-Saharan language, cluster further away from the Bt20+ samples, as would anticipated. The south-western Bantu-speakers (SWB), cluster relatively closer to the Bt20+ group and also speak a Niger-Congo languages. Results from PCA show that language (based on self-identification) has a high correlation with genetic variation. This correlation has been noted in other ancestral studies of African population groups (1, 3-6).

Extract SNPs

Birth to Twenty Plus cohort (127764 Metabochip SNPs)

(N=971 Black Africans)

Locke et al. (97 adult BMI SNPs)

(N=339224 predominantly Europeans)

19 SNPs not found

78 SNPs extracted

Check proxy SNPs using SNAP[[1](#_ENREF_1)]

9 SNPs dropped:

1 triallelic, 8 homozygous SNPs

2 proxy SNPs found using Yoruba, Luhya and Maasai as reference populations

69 SNPs remain

71 adult BMI SNPs remain for genetic risk score construction

**References:**

1. Johnson AD, Handsaker RE, Pulit SL, et al. SNAP: a web-based tool for identification and annotation of proxy SNPs using HapMap. Bioinformatics 2008;**24**(24):2938-39

**Figure S2: Flowchart displaying the selection of BMI related SNPs for inclusion in the weighted BMI genetic risk score**


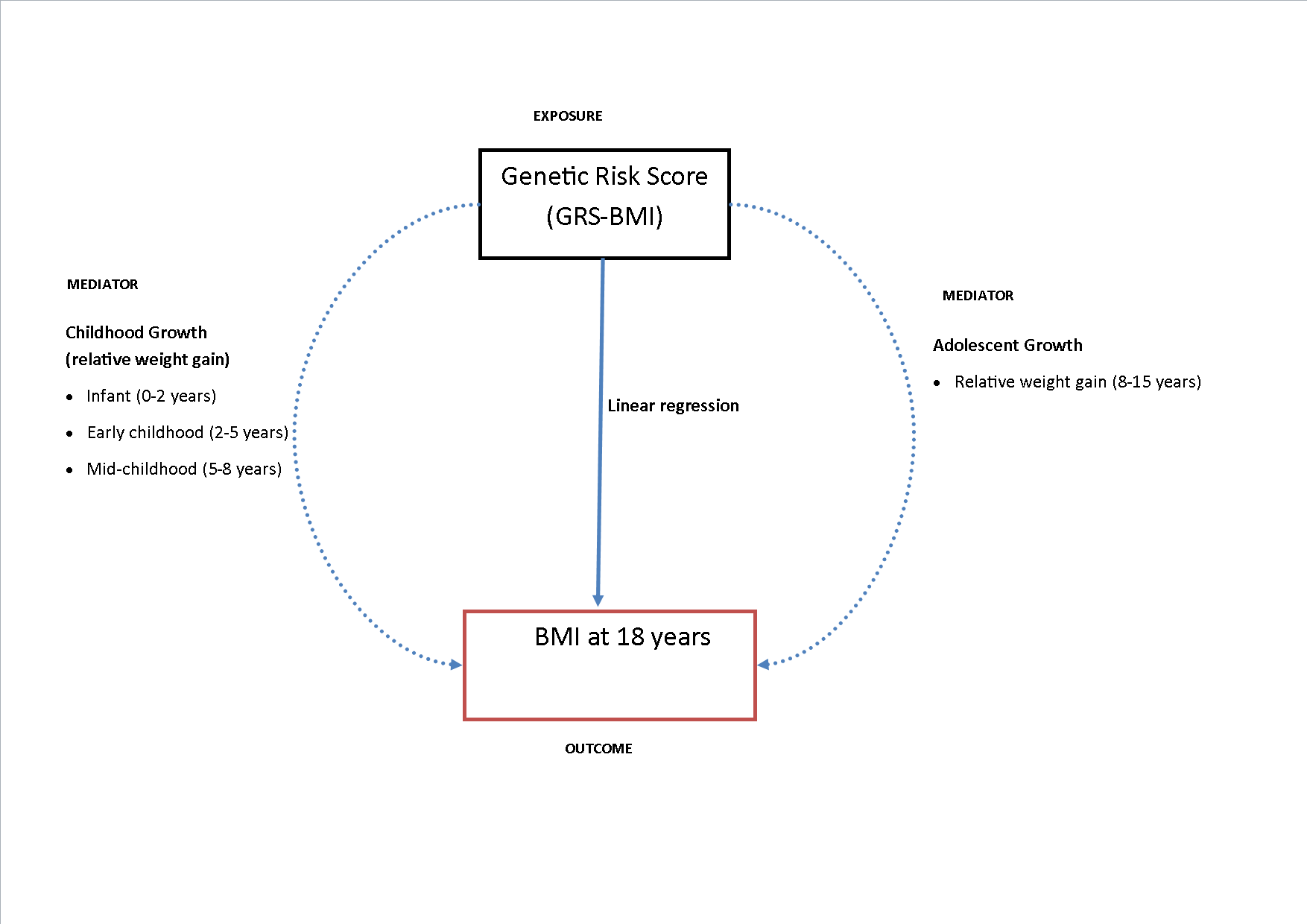


**Figure S3: Schematic diagram for the mediation analysis to assess whether the association between wGRS and obesity risk at 18 years was mediated by growth**

**
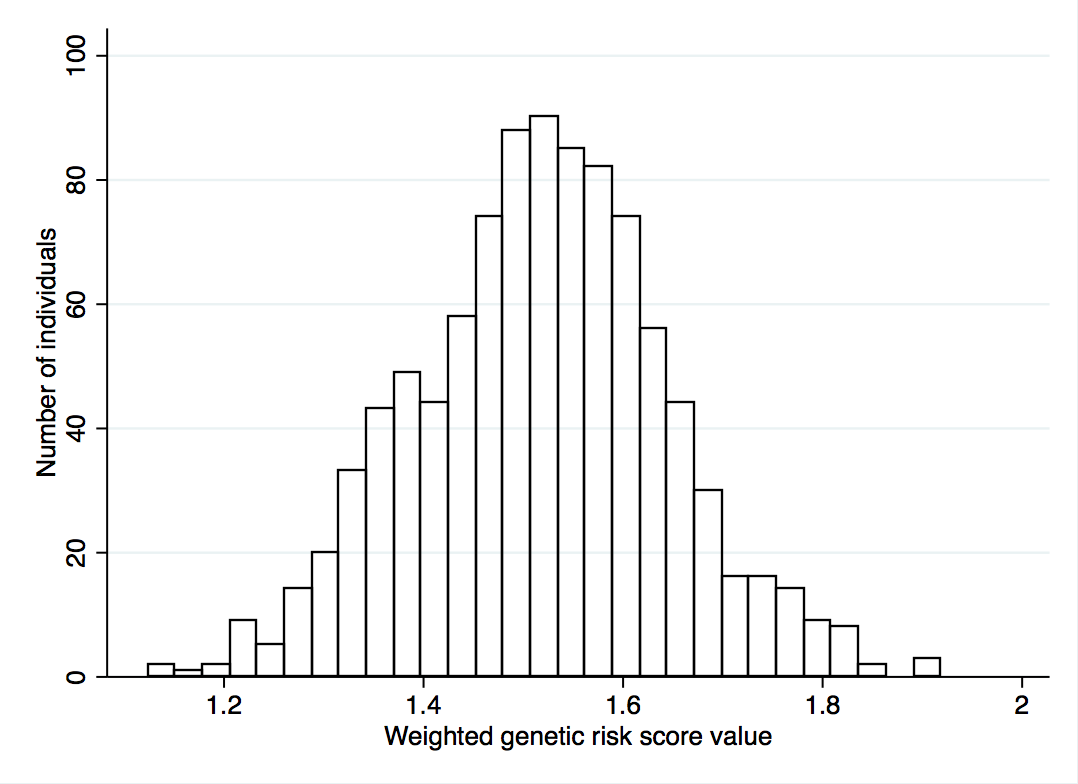
**

**Figure S4:** Distribution of the weighted genetic risk score for adult BMI in the Birth to

Twenty Plus cohort.


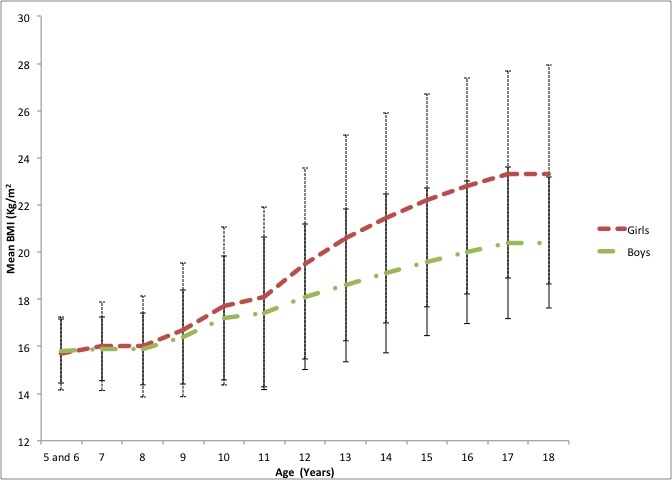


**Figure S5**: The average BMI (standard deviation bars) values from 5 to 18 years in the Birth to

Twenty Plus cohort

| **Table S2: Path coefficients, direct and total effect estimates: The role of conditional relative weight gain as a mediator of the association between the weighted genetic risk score (wGRS) and BMI at 18 years of age**   \|  \|  \| **Paths of association (p-value)** \| \| \| \|  \| \| --- \| --- \| --- \| --- \| --- \| --- \| --- \| \| **Weight gain period** \| **Sample Size (n)** \| **^a^wGRSz to Weight Gain** \| **^b^Weight Gain to BMI at 18 years** \| **^c^wGRSz to BMI at 18 years**  **(total effect)** \| **wGRSz to BMI at 18 years**  **(direct effect)** \| **^d^Proportion mediated by** \| \| Infancy  (0-2 years) \| 630 \| 0.007 \| 1.147** \| 0.460** \| 0.452** \| 0.017 \| \| Early childhood (2-5 years) \| 574 \| -0.027 \| 0.884** \| 0.435** \| 0.459** \| -0.055 \| \| Mid-childhood (5-8 years) \| 452 \| 0.067 \| 1.351** \| 0.329* \| 0.238 \| 0.28 \| \| Adolescence (8-15 years) \| 514 \| 0.091* \| 2.160** \| 0.347* \| 0.151 \| 0.56 ^*@^ \|   *P < 0.05 uncorrected; **P < The Holm correction significance; ^*@^Sobel test of mediation P < 0.05 uncorrected; ^*^  ^*@^Sobel test of mediation P < Holm corrected significant level.    **Notes:** ^a^wGRSz to Weight Gain is measured as standardized residuals (SR) of weight gain per SD of wGRSz  ^b^Weight Gain to BMI is measured as BMI SDS per SR of weight gain  ^c^wGRSz to BMI at 18 is measured as BMI SDS per SD of wGRSz  ^d^Proportion of the total wGRSz to BMI at 18 association that is mediated by conditional relative weight gain  **Table S3: Association between the 71 SNPs and BMI at 18 years** | | | | | | | |
| --- | --- | --- | --- | --- | --- | --- | --- | --- | --- | --- | --- | --- | --- | --- | --- | --- | --- | --- | --- | --- | --- | --- | --- | --- | --- | --- | --- | --- | --- | --- | --- | --- | --- | --- | --- | --- | --- | --- | --- | --- | --- | --- | --- | --- | --- | --- | --- | --- | --- |
| **SNP** | **Chr.** | **Position (bp/hg19)** | **Nearest gene** | **Risk/effect Allele** | **p-Value** | **beta ( kg/m^2^)** | **95% CI ( kg/m^2^)** |
| rs1000940 | 17 | 5,223,976 | *RABEP1* | G | 0.53 | -0.12 | [-0.54; 0.28] |
| rs10408163(1) | 19 | 52,260,843 | *ZC3H4* | A | 0.37 | -0.28 | [-0.90; 0.34] |
| rs10733682 | 9 | 128,500,735 | *LMX1B* | A | 0.85 | 0.05 | [-0.42; 0.51] |
| rs10968576 | 9 | 28,404,339 | *LINGO2* | G | 0.66 | -0.12 | [-0.66; 0.40] |
| rs11126666 | 2 | 26,782,315 | *KCNK3* | A | 0.78 | -0.07 | [-0.53; 0.4] |
| rs11583200 | 1 | 50,332,407 | *ELAVL4* | C | 0.29 | 0.21 | [-0.18; 0.59] |
| rs1167827 | 7 | 75,001,105 | *HIP1* | G | 0.74 | -0.11 | [-0.75; 0.53] |
| rs11688816 | 2 | 62,906,552 | *EHBP1* | G | 0.77 | -0.05 | [-0.41; 0.31] |
| rs11847697 | 14 | 29,584,863 | *PRKD1* | T | 0.85 | -0.03 | [-0.41; 0.34] |
| rs12286929 | 11 | 114,527,614 | *CADM1* | G | 0.41 | 0.15 | [-0.21; 0.52] |
| rs12401738 | 1 | 78,219,349 | *FUBP1* | A | 0.61 | 0.2 | [-0.57; 0.98] |
| rs12446632 | 16 | 19,842,890 | *GPRC5B* | G | 0.41 | 0.27 | [-0.36; 0.90] |
| rs12566985 | 1 | 74,774,781 | *FPGT-TNNI3K* | G | 0.19 | 0.28 | [-0.41; 0.34] |
| rs12940622 | 17 | 76,230,166 | *RPTOR* | G | 0.61 | 0.1 | [-0.28; 0.48] |
| rs13021737 | 2 | 622,348 | *TMEM18* | G | 0.39 | 0.29 | [-0.38; 0.95] |
| rs13191362 | 6 | 162,953,340 | *PARK2* | A | 0.38 | -0.3 | [-0.96; 0.37] |
| rs1441264 | 13 | 78,478,920 | *MIR548A2* | A | 0.92 | 0.02 | [ -0.37; 0.41] |
| rs1460676 | 2 | 164,275,935 | *FIGN* | C | 0.85 | 0.04 | [-0.39; 0.48] |
| rs1516725 | 3 | 187,306,698 | *ETV5* | C | 0.38 | 0.19 | [-0.23; 0.61] |
| rs1528435 | 2 | 181,259,207 | *UBE2E3* | T | 0.37 | 0.18 | [-0.21; 0.57] |
| rs1558902 | 16 | 52,361,075 | *FTO* | A | 0.15 | -0.6 | [ -1.40; 0.21] |
| rs16907751 | 8 | 81,538,012 | *ZBTB10* | C | 0.72 | 0.1 | [-0.47; 0.68] |
| rs16951275 | 15 | 65,864,222 | *MAP2K5* | T | 0.04 | 0.4 | [ 0 .02; 0.79] |
| *rs17001561(2)* | 4 | 77,348,592 | *SCARB2* | G | 0.21 | 0.34 | [-0.20; 0.88] |
| rs17024393 | 1 | 109,956,211 | *GNAT2* | C | 0.86 | 0.05 | [-0.53; 0.63] |
| rs17405819 | 8 | 76,969,139 | *HNF4G* | T | 0.66 | 0.16 | [ -0.56; 0.89] |
| rs17724992 | 19 | 18,315,825 | *PGPEP1* | A | 0.34 | 0.42 | [-0.44; 1.29] |
| rs1808579 | 18 | 19,358,886 | *C18orf8* | C | 0.006 | 0.53 | [0.15; 0.91] |
| rs1928295 | 9 | 119,418,304 | *TLR4* | T | 0.98 | 0.004 | [ -0.38; 0.39] |
| rs2033732 | 8 | 85,242,264 | *RALYL* | C | 0.6 | 0.21 | [ -0.57; 0.98] |
| rs205262 | 6 | 34,671,142 | *C6orf106* | G | 0.33 | 0.21 | [ -0.21; 0.62] |
| rs2075650 | 19 | 50,087,459 | *TOMM40* | A | 0.4 | 0.21 | [-0.28; 0.71] |
| rs2080454 | 16 | 47,620,091 | *CBLN1* | C | 0.22 | 0.24 | [-0.14; 0.62] |
| rs2112347 | 5 | 75,050,998 | *POC5* | T | 0.61 | -0.1 | [ -0.46; 0.21] |
| rs2121279 | 2 | 142,759,755 | *LRP1B* | T | 0.8 | -0.09 | [-0.79; 0.61] |
| rs2176040 | 2 | 226,801,046 | *LOC646736* | A | 0.44 | -0.16 | [-0.56; 0.24] |
| rs2176598 | 11 | 43,820,854 | *HSD17B12* | T | 0.48 | 0.14 | [-0.25; 0.54] |
| rs2207139 | 6 | 50,953,449 | *TFAP2B* | G | 0.23 | 0.46 | [-0.29; 1.20] |
| rs2245368 | 7 | 76,446,079 | *PMS2L11* | C | 0.41 | 0.18 | [-0.25; 0.62] |
| rs2287019 | 19 | 50,894,012 | *QPCTL* | C | 0.55 | 0.18 | [-0.40; 0.76] |
| rs2365389 | 3 | 61,211,502 | *FHIT* | C | 0.99 | -0.0004 | [-0.52; 0.52] |
| rs2820292 | 1 | 200,050,910 | *NAV1* | C | 0.73 | 0.07 | [-0.31; 0.44] |
| rs2836754 | 21 | 39,213,610 | *ETS2* | C | 0.17 | 0.32 | [-0.13; 0.76] |
| rs29941 | 19 | 39,001,372 | *KCTD15* | G | 0.92 | -0.03 | [-0.55; 0.50] |
| rs3101336 | 1 | 72,523,773 | *NEGR1* | C | 0.004 | 0.54 | [0.18; 0.89] |
| rs3736485 | 15 | 49,535,902 | *DMXL2* | A | 0.55 | -0.12 | [-0.53; 0.28] |
| rs3817334 | 11 | 47,607,569 | *MTCH2* | T | 0.36 | -0.19 | [-0.59; 0.22] |
| rs3888190 | 16 | 28,796,987 | *ATP2A1* | A | 0.2 | 0.26 | [-0.14; 0.66] |
| rs4256980 | 11 | 8,630,515 | *TRIM66* | G | 0.4 | -0.16 | [-0.54; 0.22] |
| rs4740619 | 9 | 15,624,326 | *C9orf93* | T | 0.65 | 0.08 | [-0.28; 0.45] |
| rs4787491 | 16 | 29,922,838 | *INO80E* | G | 0.28 | -0.2 | [-0.56; 0.16] |
| rs543874 | 1 | 176,156,103 | *SEC16B* | G | 0.29 | 0.23 | [-0.20; 0.67] |
| rs6465468 | 7 | 95,007,450 | *ASB4* | T | 0.88 | 0.05 | [-0.58; 0.68] |
| rs6477694 | 9 | 110,972,163 | *EPB41L4B* | C | 0.37 | 0.17 | [-0.21; 0.55] |
| rs6567160 | 18 | 55,980,115 | *MC4R* | C | 0.1 | 0.41 | [-0.08; 0.89] |
| rs657452 | 1 | 49,362,434 | *AGBL4* | A | 0.41 | 0.15 | [-0.21; 0.52] |
| rs6804842 | 3 | 25,081,441 | *RARB* | G | 0.77 | 0.06 | [-0.35; 0.48] |
| rs7138803 | 12 | 48,533,735 | *BCDIN3D* | A | 0.8 | -0.06 | [-0.55; 402] |
| rs7141420 | 14 | 78,969,207 | *NRXN3* | T | 0.83 | -0.04 | [-0.40; 0.32] |
| rs7164727 | 15 | 70,881,044 | *LOC100287559* | T | 0.03 | -0.45 | [-0.85; -0.04] |
| rs7239883 | 18 | 38,401,669 | *LOC284260* | G | 0.71 | -0.07 | [-0.43; 0.29] |
| rs7243357 | 18 | 55,034,299 | *GRP* | T | 0.45 | -0.22 | [-0.78; 0.35] |
| rs7599312 | 2 | 213,121,476 | *ERBB4* | G | 0.22 | 0.24 | [-0.14; 0.62] |
| rs7715256 | 5 | 153,518,086 | *GALNT10* | G | 0.44 | 0.15 | [-0.23; 0.52] |
| rs7899106 | 10 | 87,400,884 | *GRID1* | G | 0.24 | 0.35 | [-0.23; 0.92] |
| rs7903146 | 10 | 114,748,339 | *TCF7L2* | C | 0.14 | 0.28 | [-0.09; 0.66] |
| rs9374842 | 6 | 120,227,364 | *LOC285762* | T | 0.2 | 0.31 | [-0.16; 0.78] |
| rs9400239 | 6 | 109,084,356 | *FOXO3* | C | 0.9 | 0.03 | [-0.45; 0.51] |
| rs977747 | 1 | 47,457,264 | *TAL1* | T | 0.51 | 0.14 | [-0.27; 0.54] |
| rs9914578 | 17 | 1,951,886 | *SMG6* | G | 0.39 | 0.17 | [-0.27; 0.55] |
| rs9925964 | 16 | 31,037,396 | *KAT8* | A | 0.03 | -0.78 | [-1.5; -0.07] |

**References:**

1. Li S, Schlebusch C, Jakobsson M. Genetic variation reveals large-scale population expansion and migration during the expansion of Bantu-speaking peoples. Proceedings Biological sciences / The Royal Society. 2014;281(1793).

2. Wood ET, Stover DA, Ehret C, Destro-Bisol G, Spedini G, McLeod H, et al. Contrasting patterns of Y chromosome and mtDNA variation in Africa: evidence for sex-biased demographic processes. European journal of human genetics : EJHG. 2005;13(7):867-76.

3. 1000 Genomes Project C. A map of human genome variation from population-scale sequencing. Nature. 2010;467(7319):1061-73.

4. May A, Hazelhurst S, Li Y, Norris SA, Govind N, Tikly M, et al. Genetic diversity in black South Africans from Soweto. BMC genomics. 2013;14:644.

5. Nettle D, Harriss L. Genetic and linguistic affinities between human populations in Eurasia and West Africa. Human biology. 2003;75(3):331-44.

6. Schlebusch CM, Skoglund P, Sjodin P, Gattepaille LM, Hernandez D, Jay F, et al. Genomic variation in seven Khoe-San groups reveals adaptation and complex African history. Science. 2012;338(6105):374-9.
